# CD40L promotes development of acute aortic dissection via induction of inflammation and impairment of endothelial cell function

**DOI:** 10.18632/aging.101394

**Published:** 2018-03-04

**Authors:** Lu Han, Lu Dai, Yuan-Fei Zhao, Hai-Yang Li, Ou Liu, Feng Lan, Wen-Jian Jiang, Hong-Jia Zhang

**Affiliations:** 1 Department of Cardiac Surgery, Beijing Anzhen Hospital, Capital Medical University, Beijing, China; 2 Centre for Transplant and Renal Research, The Westmead Institute for Medical Research, University of Sydney, Sydney, Australia; 3 Beijing Institute of Heart, Lung and Blood Vessel Diseases, Beijing, China; 4 Beijing Lab for Cardiovascular Precision Medicine, Beijing, China; 5 Beijing Aortic Disease Center, Cardiovascular Surgery Center, Beijing, China; 6 Beijing Engineering Research Center for Vascular Prostheses, Beijing, China; * Equal contribution

**Keywords:** CD40L, acute aortic dissection, endothelial cell

## Abstract

Acute aortic dissection is one of the most lethal cardiovascular disease. The major histopathological feature of AAD is medial degradation, especially breakdown of elastin and collagen. However, the underlying mechanism remains a mystery. Platelets expressed CD40 Ligand (CD40L) is recently recognised as a key effector of cardiovascular disease development through its pro-inflammatory effect. To clarify the role of CD40L in AAD, we examined level of CD40L in human blood serum samples and found that it is significantly higher in AAD patients compared with healthy subjects (26.8±5.52 ng/mL versus 13.4±4.00 ng/mL). To further investigate if CD40L is involve in the development of AAD, we applied β-aminopropionitrile (BAPN) induced mouse model of AAD. Consistent with the human data, circulating CD40L in AAD mice much higher than normal mice (148.40±75.96 pg/mL versus 44.09±19.65 pg/mL). Meanwhile, multiple pro-inflammatory chemokines significantly increased in AAD mice. Importantly, the CD40L-/- mice treated with BAPN did not develop these phenotypes. Lastly, we confirmed that endothelial cells migration was significantly inhibited by CD40L, suggesting impaired recovery from intimal injury. In summary, we found that CD40L promoted AAD development through its pro-inflammatory effects and inhibition of endothelial cell function.

## Introduction

Acute aortic dissection (AAD) accounts for a substantial part of deaths from aortic diseases [1]. Type A AAD is the most complicated form according to Stanford classification, which is characterized by abrupt onset, rapid progression and poor prognosis. Type A AAD has a mortality of 50% within the first 48 hours and 90% within 1 month if not operated. However, cumulative evidence has proved that surgery can reduce 1-month mortality to 30% or even lower [2]. Hence, a biomarker which is sensitive and specific for diagnosis or a therapeutic target for delaying development of AAD is of great importance. Elucidation of the risk factors and underlying pathological mechanisms associated with the disease will help us achieve that goal.

Pathophysiologically, aortic dissection is defined as disruption of the medial layer provoked by intramural bleeding, resulting in separation of the aortic wall layers and subsequent formation of a true lumen (TL) and a false lumen (FL) with or without communication [2]. Taking account of the histopathological findings, medial degeneration is the most widely accepted risk factor that induces AAD formation and is described as disruption and loss of elastic fibers and increased deposition of proteoglycans [1,3]. Thus, medial integrity maintained by collagen and elastin cross-links is one of the key points to protect from AAD. β-aminopropionitrile (BAPN) is described as a lysyl oxidase inhibitor, which cause defective elastin and collagen synthesis and create a pre-AAD status in immature mice [4,5]. Mice fed with BAPN has been reported to suffer from AAD and is successfully established as an AAD model with a successful rate of over 80% [3,6,7].

However, AAD is most often initiated by intimal tear, resulting in communication of the two lumens and further disruption of vessel wall [2]. That makes us wonder if intimal injury or inflammation is also a risk factor which participate in the development of AAD. CD40 ligand (CD40L, CD154), an intrinsic membrane glycoprotein which constitutes a member of the TNF ligand superfamily, has been a star molecule of cardiovascular research since 1998 and is largely expressed by platelets [8–10]. Originally regarded as a key role in cellular immunity, it has now been proved to induce atherosclerosis and thrombosis through its pro-inflammatory effects [11]. CD40 is the main receptor that interacted with CD40L and is widely expressed by many cells, including endothelial cells [9]. Our previous work has revealed that platelet is involved in the development of AAD [12]. Hence, the aim of this experiment is to investigate whether platelet expressed CD40L is able to impair endothelial cells and further induce AAD formation.

## RESULTS

### Elevated levels of CD40L, pro-inflammatory chemokines and adhesion molecules in blood samples from AAD patients

We first compared demographic factors in healthy volunteers and type A AAD patients, there were no significant differences in average age and ratio of men to women among these groups (Table 1). We then screened the levels of circulating CD40L in blood samples from healthy volunteers and patients with type A AAD, the levels were significantly higher in all patients compared with healthy volunteers (Table 1). CD40L-CD40 interaction is known to up-regulate adhesion molecules (e.g. E-selectin and VCAM-1 [vascular cell adhesion molecule 1]), pro-inflammatory chemokines (e.g. IL-6, TNF-α [tumor necrosis factor-α] and MCP-1 [monocyte chemoattractant protein-1]) as well as MMPs (MMP-2 and -9) [8,10]. Thus, we also measured the IL-1β, IL-6, TNF-α, VCAM-1, E-selectin, MCP-1, MMP-2 and MMP-9 levels in serum samples of different groups, and found that the circulating levels of them all were significantly higher in the type A AAD group than the healthy volunteers group studied (Table 1).

**Table 1:** Background and cytokine of human peripheral blood.

	AAD(100)	Control(139)	P value
Male (%)	70(70)	98(70.5)	0.933
YearD)	48.3±11.97	48.8±9.98	0.718
BMI	25.3±3.71	24.2±3.45	0.017
Hypertension (%)%	44(44)	24(17.3)	<0.001
sCD40L (ng/mL)	26.8±5.52	13.4±4.00	<0.001
E-Selectin (ng/mL)	116.1±24.88	77.1±14.30	<0.001
IL-1β (pg/mL)	62.6±13.28	48.6±9.76	<0.001
IL-6 (pg/mL)	500.8±91.61	421.4±33.87	<0.001
MCP-1 (pg/mL)	217.9±56.22	140.8±28.07	<0.001
MMP-2 (ng/mL)	196.0±34.43	168.4±13.34	<0.001
MMP-9 (ng/mL)	469.1±77.74	326.0±30.51	<0.001
VCAM-1 (ng/mL)	516.2±86.53	396.9±57.41	<0.001
TNF-α (pg/mL)	55.6±9.04	37.1±5.43	<0.001

BMI indicates body mass index; AAD, acute aortic dissection.

### Significantly higher mortality and prevalence rate of AAD in wild mice treated with BAPN

**Figure 1:**
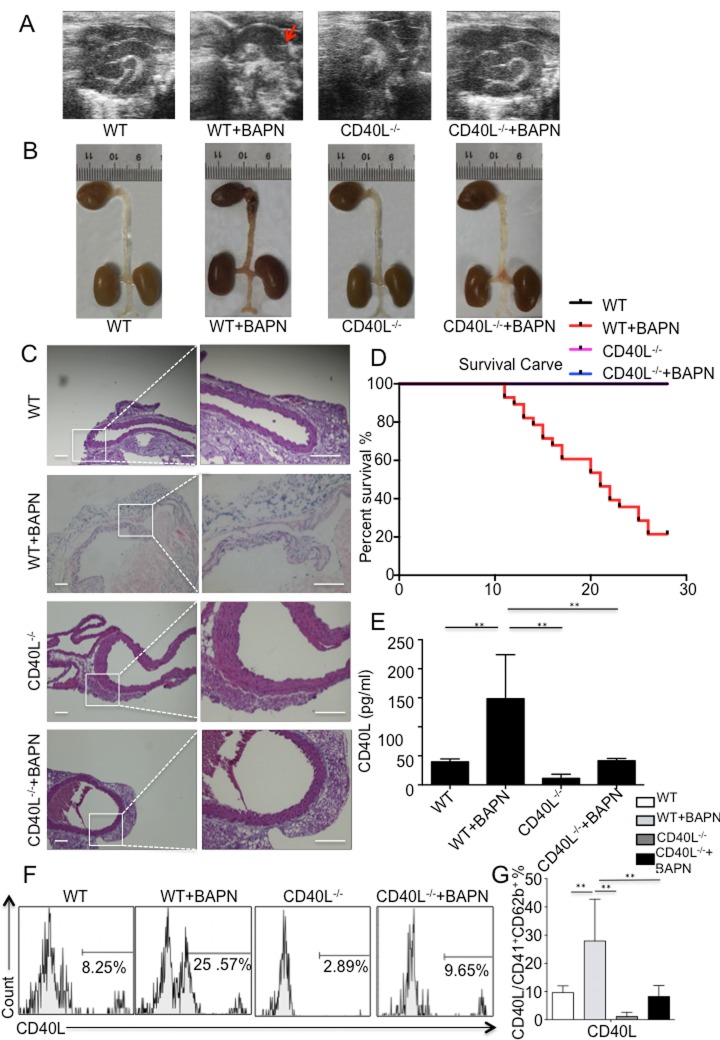
**BAPN administration-induced TAD formation is inhibited by genetic depletion of CD40L.** (**A**) Echography showed aortic arch dilation. (**B**) Representative images showed autopsy features of isolated mouse aorta after feeding with BAPN or saline for 28 days; arrow, location of TAD. (**C**) Haematixylin and eosin (H&E) staining showed significant dissected intima in WT+BAPN mice, while flat intima could be seen in other 3 groups. (**D**) Survival curve of WT (n=12), WT+BAPN (n=27), CD40L-/- (n=12) and CD40L-/-+BAPN (n=17) mice. (**E**) ELISA of sCD40L showed that circulating levels of CD40L are elevated in plasma from WT+BAPN mice. (**F**) Representative flow cytometry analysis showed percentage of CD41+ CD62b+ CD40L+ platelets divided by total CD41+ CD62b+ platelets in mice plasma. (**G**) Statistical data analysis from four separate experiments. Data were expressed as percentage ± SD of the CD41+ CD62b+ CD40L+ platelets subpopulations in total CD41+ CD62b+ platelets counted. **P < 0.01 versus WT, CD40L-/- and CD40L-/-+BAPN mice.

All 3-week-old mice were divided into 2 groups. Group A consisted of 15 mice and all mice were treated with water. Group B consisted of 30 mice and all mice were treated with BAPN solution. During the experiment, mice were monitored using echography, and aortic dilation could be detected in group B (Fig. 1A) without high-resolution imaging of intimal flap. Till the end of the experiment, 21 mice in group B died, 16 of which were diagnosed with thoracic aortic dissection (TAD) after autopsy (Fig. 1B), other 5 possessing normal aorta. 9 surviving mice were sacrificed for flow cytometry, histology and analysis of plasma. For group A, all mice were alive until the end of experiment and all of them were sacrificed for flow cytometry, histology and analysis of plasma.

Haematixylin and eosin (H&E) staining showed dissected intima and disruption of media on the location of thoracic aorta after BAPN administration (Fig. 1C).

Eventually, except for 3 mice sacrificed for flow cytometry without autopsy, 21 were diagnosed with AAD in group B, indicating a successful rate of 77.78% (Fig. 1D).

### Platelet expressed CD40L correlates with elevation of pro-inflammatory chemokines in BAPN treated wild mice

Blood samples were taken from all survival mice in 2 groups, and circulating CD40L levels were detected using ELISA. We could tell from the result that level of CD40L was significantly higher in group B than group A (Fig. 1E). Since activated platelets are major source of membrane bound CD40L and soluble CD40L, and our previous work have proved that platelets are largely activated during the process of aortic dissection formation, then we further investigate the amount of platelets expressed CD40L in 2 groups through flow cytometry. Analysis data demonstrated that there were more platelets expressed CD40L in group B compared with group A (Fig. 1F, 1G).

**Figure 2:**
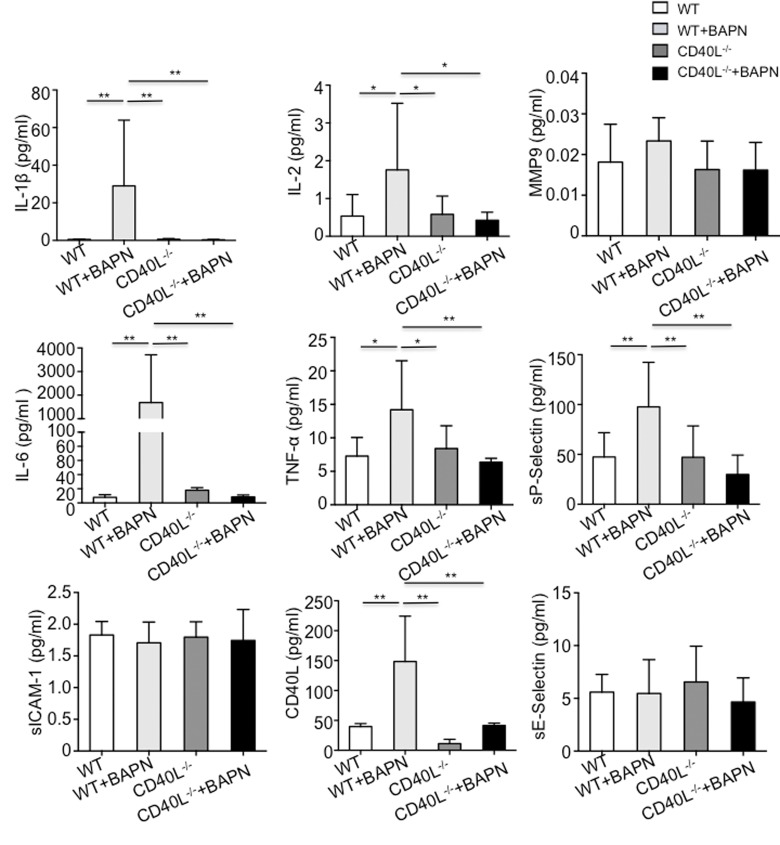
**Circulating pro-inflammatory chemokines were elevated in plasma of WT+BAPN mice.** E-selectin, ICAM-1, P-selectin, IL-1β, IL-2, IL-6, TNF-α and MMP-9 were examined. IL-1β, IL-2, IL-6 and TNF-α and P-selectin were significantly higher in WT+BAPN mice. Values are mean ±SEM. *P<0.05 versus other groups, **P<0.01 versus other groups. MMP9 indicates matrix metalloproteinase 9; ICAM-1, intercellular cell adhesion molecule-1.

To clarify whether pro-inflammatory chemokines and endothelial adhesion molecules were released after interaction of CD40L with endothelial cells expressed CD40, many biomarkers including E-selectin, ICAM-1, P-selectin, IL-1β, IL-2, IL-6, TNF-α and MMP-9 were tested. Quantitative results implicated that levels of IL-1β, IL-2, IL-6 and TNF-α and P-selectin were significantly higher in group B. However, endothelial adhesion molecules such as E-selectin and ICAM-1 did not differ between the 2 groups. Also we found no differences in MMP-9 levels (Fig. 2).

### Prevention of AAD by genetic depletion of CD40L

To determine whether CD40L plays a key role in mice treated with BAPN, CD40L^-/-^ mice was obtained and fed in the same way as we did to wild mice. In brief, CD40L^-/-^ mice was divided into 2 groups, which is group C (n=15) and group D (n=20). Mice in group D were treated with BAPN and mice in group C were not. To our surprise, neither in group C nor group D, there were no mice died before the end of experiment. Consistent with our observation, we did not detect aortic lesion during the experiment using echography and autopsy confirmed that (Fig. 1A, 1B). Haematixylin and eosin (H&E) staining exhibited normal aortic wall, without dissected intima and disruption of media (Fig. 1C). All mice in group C and group D survived after 28 days (Fig. 1D). In respect of all pro-inflammatory chemokines and endothelial adhesion molecules we’ve listed before, there were no differences between 2 groups. However, when compared with group B, plasma levels of IL-1β, IL-2, IL-6 and TNF-α and P-selectin in group C and group D was significantly lower while others were similar (Fig. 2).

### CD40L inhibited endothelial cell migration

**Figure 3:**
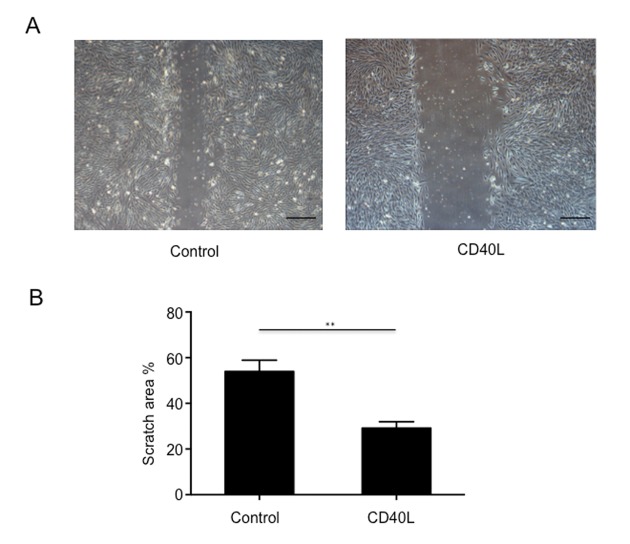
**Endothelial cells migration was significantly inhibited by CD40L.** (**A**) Representative images showed different scratch area in groups incubated with or without recombinant human soluble CD40 ligand. (**B**) Quantitative analysis showed significantly larger scratch area in wound area incubated with CD40L. Data were expressed as percentage ± SD. **P < 0.05.

In our exploration of CD40L on endothelial cells, HUVECs were obtained and cultured with or without CD40L, and endothelial cells migration were assessed by a scratched wound assay. The results showed that scratch area was 53.97±2.846% in group 1 and 29.14±1.645% in group 2 (Fig. 3A, 3B). We could see from the image that CD40L significantly inhibited endothelial cells migration.

## DISCUSSION

AAD is reported to be one of the most fatal cardiovascular disease [13–15]. Thus, rapid diagnosis and effective treatment is essential. Computed Tomography Angiography (CTA) is known to be the gold standard for diagnosis. However, it is not practical and cheap to perform CTA to every patient complaining with chest pain. A biomarker with high sensitivity and specialty that can warn clinicians of AAD is of great importance. Until now, many biomarkers have been tested [16]. Of them all, only d-dimer has a clinically relevant role in the setting of suspected aortic dissection. In our current experiment, we have elucidated that serum CD40L was significantly higher in type A AAD patients, providing a potential biomarker for AAD diagnosis [17]. However, we did not test CD40L level in type B AAD. The sensitivity and specialty of CD40L in diagnosing AAD remain to be demonstrated.

Due to its insidiousness in regard to formation and its acuteness in regard to onset, underlying pathological mechanisms responsible for AAD development remain elusive. Fortunately, we have successfully established an AAD model which is beneficial for us to learn more about AAD [6,7]. Indeed, BAPN was previously used to establish aortic aneurysm model and we did not know whether there has been aortic dissection or not. In recent years, some researchers have established AAD model using BAPN treatment companying with Ang II stimulation. Tomohiro Kurihara et al have reported a successful rate of 100% and Atsushi Anzai et al have reported a successful rate of 70% [3,5]. Our previous applications of BAPN alone treatment have reported successful rate of 87% and 89% respectively. Hence, the BAPN model is a reliable model for us to investigate AAD.

At present, platelets are acknowledged to interface with thrombosis, inflammation, and immunity [18–20]. Interestingly, it was not until CD40L was demonstrated to be largely expressed by platelets in 1998 that platelets were admitted to participate in inflammation reaction [9]. Since then, CD40L have been a key effector in the interaction of platelets and cardiovascular diseases. The relationship between coronary artery disease (CAD) and CD40L is well studied. For example, CD40L is proved to increase neointimal formation after arterial injury and is proved to activate platelets and further trigger an inflammatory reaction of endothelial cells [21–23]. Moreover, endothelial dysfunction was previously elucidated to play a role in AAD development by endoplasmic reticulum (ER) stress dependent microparticles derived from smooth muscle cells [24]. However, to our knowledge, no one have ever investigated the effects of CD40L on the endothelial cells of aorta. In our study, BAPN-induced AAD could be inhibited by CD40L gene knock out and in vitro study showed suppression of endothelial cell migration. Thus, we are the first to correlate CD40L with endothelial cells dysfunction in the development of AAD. Besides, among all the biomarkers that indicate the injury of endothelial, EMP (Endothelial microparticles) is the one of the most widely accepted [25,26]. However, no single marker for EMP exists, as many antigens are also present on a number of different cell sub-sets [27]. Thus, we tested many pro-inflammatory chemokines and endothelial adhesion molecules which have been validated to be markers of endothelial cells CD40L-CD40 interaction in order to identify endothelial cells dysfunction. Of them, VCAM-1, E-selectin, P-selectin, MCP-1, IL-6, MMP-2 and MMP-9 were previously demonstrated to promote AAD formation [3,5,28–31]. DU XM et al observed TNF-α mRNA was elevated in blood samples of AAD patients [32]. Weis-Müller BT et al reported that IL-2 gene expression was up-regulated in the lesion of AAD [33]. And not surprisingly, TNF-α and IL-2 plasma levels were found significantly higher [34]. With regard to IL-1β and ICAM-1, Jia LX et al confirmed larger expression of mRNA in aorta specimens of AAD patients and mouse [24]. In addition, ICAM-1 has been identified to be a risk factor for spontaneous cervical artery dissection [35]. In the present study, IL-1β, IL-6, TNF-α, VCAM-1, E-selectin, MCP-1, MMP-2 and MMP-9 levels in human serum samples were identical to studies performed by other researchers. Nevertheless, what we found in mouse plasma samples were a little different, E-selectin and ICAM-1 and MMP-9 did not differ between AAD mouse and wild mouse, possibly suggest other mechanisms in the process of AAD formation.

This study has a few limitations. First, except for platelets, CD40L was reported to be expressed by many other cells. The contribution of CD40L from other cells in peripheral blood need further investigation. Second, we did not perform immunohistochemical analysis on aortic specimens from AAD patients and mice, so we did not demonstrate that there is neutrophil infiltration on aortic wall after AAD formation. However, previous studies have shown that there is neutrophil infiltration in dissected aorta [3,5]. Third, we did not regularly test levels of CD40L and other factors during the 28 experimental days, which is hard for us to judge whether CD40L continuously contribute to the whole progress of the BAPN-induced development of Acute Aortic Dissection or just certain stage. Last, most experiments were performed in mice, so whether anti-CD40L therapy is a potential medical treatment target needs to be investigated by future clinical study.

## CONCLUSION

CD40L induces endothelial cells dysfunction through pro-inflammatory effect and further promote AAD development. Thus, CD40L is a potential biomarker for diagnosis and medical treatment to reduce the risk of AAD.

## MATERIALS AND METHODS

### Human blood samples

Between August 2016 and February 2017, 100 patients diagnosed with type A AAD and 139 healthy volunteers were registered in the study. All the patients were free from connective tissue disorders such as Marfan syndrome, Ehlers-Danlos syndrome, and aortitis diagnosed according to the clinical history and physical examinations. The blood samples from patients were collected within 1 hour before surgery. Diagnosis of AAD was confirmed by computed tomography angiography. The blood samples were collected in the Beijing Anzhen Hospital, and signed informed consent for the usage of the samples in the experiments. This study was approved by Medical Ethical Committee of Capital Medical University, Beijing, and complied with the principles outlined in the Declaration of Helsinki [7].

### Establishment of AAD model in mice

Wild-type (WT) and CD40L^-/-^ mice on C57BL/6 background were purchased from Jackson Laboratory (Bar Harbor, ME) and gene knocking out of CD40L were validated (see Supplementary Material). Three-week old male mice were fed on a regular diet and administered BAPN (Sigma-Aldrich, St. Louis, MO, USA) dissolved in drinking water (1 g/kg per day) for 4 weeks as described previously [6]. All mice died before expected end time of the experiment were autopsied immediately, mice surviving at the end of the experiment were sacrificed by an overdose of sodium pentobarbital and their blood and tissue samples were collected for further analyses [6]. Mice were scanned by a microcomputed tomographic system (GE Healthcare, Tokyo, Japan) for imaging of aortas. All studies were reviewed and approved by The Institutional Animal Care and Use Committee of Capital Medical University, Beijing, China.

### Aortic ultrasonography monitoring

Mice were anaesthetized with 1% isoflurane and underwent echography in M-mode, using a high-resolution micro-ultrasound system (Vevo 2100, VisualSonics, Toronto, Canada) equipped with a 30MHz transducer as previously described [7].

### Histology

Histopathological evaluations were performed with samples taken from wild mice, wild mice treated with BAPN, CD40L^-/-^ mice and CD40L^-/-^ mice treated with BAPN mice. After euthanasia, normal and dissected aortas were harvested from the ascending aorta to the iliac artery and were fixed in 10% buffered formalin, as previously described [6]. Fixed, paraffin-embedded tissues were cut at 5 μm thickness, and then stained with H&E. In each section, the aortic tissues were photographed.

### Flow cytometry

Peripheral blood of mice was obtained from eyes into an anticoagulant tube. Then blood was centrifuged at 150× g for 20 min immediately, and the supernatant was platelet rich plasma (PRP). PRP was centrifuged at 800× g for 10 min to obtain platelets. Platelets were re-suspended for use. The isolated platelets were stained with anti-CD41-FITC (Biolegend), anti-CD62P-PE (Biolegend), anti-CD40L-APC (R&D), at 4°C for 25 min in dark. To exclude nonspecific binding of antibodies to Fcγ receptors, an isotype control was set as the isolated cells were incubated with homologous nonimmune IgG (anti- Rat IgG1 (Biolegend), anti-Mouse IgG2a (Biolegend), and anti-Rat IgG2A (R&D)). Subsequently Flow cytometry was performed on a NovoCyte 3130 (ACEA, USA) and the data were analyzed with Flow Jo software (Tree Star, Ashland, OR, USA).

### Blood analysis

Mouse plasma CD40L and MMP-9 concentrations were quantified using Quantikine ELISA kits (R&D Systems) according to the manufacturer’s instructions. Analysis of IL-1β, IL-2, IL-6 and TNF-α were performed using the V-PLEX Custom Mouse Cytokine Plate (Meso Scale Discovery, Gaithersburg, MD, USA) [36,37]. The levels of E-selectin, ICAM-1 and P-selectin were detected by Milliplex Catalog MCVD1MAG77K07 Mouse CVD Mag (Millipore Corporation, St Charles, MO, USA).

Human serum CD40L, IL-1β, IL-6, TNF-α, VCAM-1, E-selectin, MCP-1, MMP-2 and MMP-9 were quantified using Quantikine ELISA kits (RZC) according to the manufacturer’s instructions.

### HUVECs culture and scratched wound assay

Pooled human umbilical vein ECs (HUVECs) were purchased from Cell Systems/Clonetics and cultured refer to previous work [38–41]. In brief, HUVEC were cultured in endothelial basal medium supplemented with hydrocortisone (1 mg/ml), bovine brain extract (3 mg/ml), gentamicin (50 mg/ml), amphotericin B (50 mg/ml), epidermal growth factor (10 mg/ml), and 10% fetal calf serum until the third passage. All HUVECs were divided into 2 groups, cells in group 1 were incubated with recombinant human soluble CD40 ligand (rh-CD40L, 0.5ug/ml, Abcam) and cells in group 2 were not. After detachment with trypsin, cells were grown for at least 18 h. Confluent monolayers of HUVEC were grown onto 6-cm wells and exposed to laminar fluid flow in a cone-and-plate apparatus as previously described. A constant shear stress of 15 dynes/cm^2^ was used in all experiments to simulate physiological shear stress.

In vitro scratched wounds were created by scraping the cell monolayer with a sterile disposable cell scraper as previously described [41–43]. To detect competence of HUVECs migration, scratch area 24 hours after injury were observed with a computer-assisted microscope (Zeiss) at 3 distinct positions (every 5 mm).

### Statistical analysis

In all experiments, data were collected and expressed as mean ± SD. Statistical analysis of the data was performed with a two-tailed Student’s t-test. P< 0.05 or P < 0.01 indicates statistical significance.

## Supplementary Material

Supplementary File
