# Correction: CD40L promotes development of acute aortic dissection via induction of inflammation and impairment of endothelial cell function

**DOI:** 10.18632/aging.101651

**Published:** 2018-11-16

**Authors:** Lu Han, Lu Dai, Yuan-Fei Zhao, Hai-Yang Li, Ou Liu, Feng Lan, Wen-Jian Jiang, Hong-Jia Zhang

**Affiliations:** 1 Department of Cardiac Surgery, Beijing Anzhen Hospital, Capital Medical University, Beijing, China; 2 Centre for Transplant and Renal Research, The Westmead Institute for Medical Research, University of Sydney, Sydney, Australia; 3 Beijing Institute of Heart, Lung and Blood Vessel Diseases, Beijing, China; 4 Beijing Lab for Cardiovascular Precision Medicine, Beijing, China; 5 Beijing Aortic Disease Center, Cardiovascular Surgery Center, Beijing, China; 6 Beijing Engineering Research Center for Vascular Prostheses, Beijing, China; * Equal contribution

Original article: Aging (Albany NY) 2018; 10: 371-385

**This article has been corrected:** The authors made a mistake in numbers of patients and volunteers in “Human blood samples” section of Materials and Methods. The correct numbers are provided below. The authors declare that this correction does not change the results or conclusions of this paper. The authors sincerely apologize for this error.

## MATERIALS AND METHODS

### Human blood samples

Between August 2016 and February 2017, **100** patients diagnosed with type A AAD and **139** healthy volunteers were registered in the study.

