# Supplementary material for: CD40L promotes development of acute aortic dissection via induction of inflammation and impairment of endothelial cell function
Source: Aging (Albany NY). 2018 Mar 4;10(3):371–85. doi: 10.18632/aging.101394 (PMC5892687; doi:10.18632/aging.101394)
Supplement: Supplementary File [file aging-10-101394-s001.pdf]

SUPPLEMENTARY MATERIAL

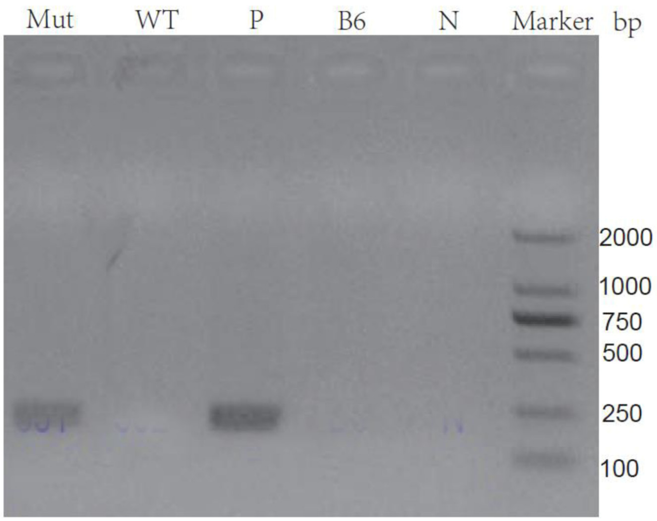

Supplementary Figure 1. PCR Identification of CD40L knockout. WT: wild-type mice; Mut: CD40L knock-out mice; P: Positive Control; B6： Negative Control; N： No-Template Control.

wild

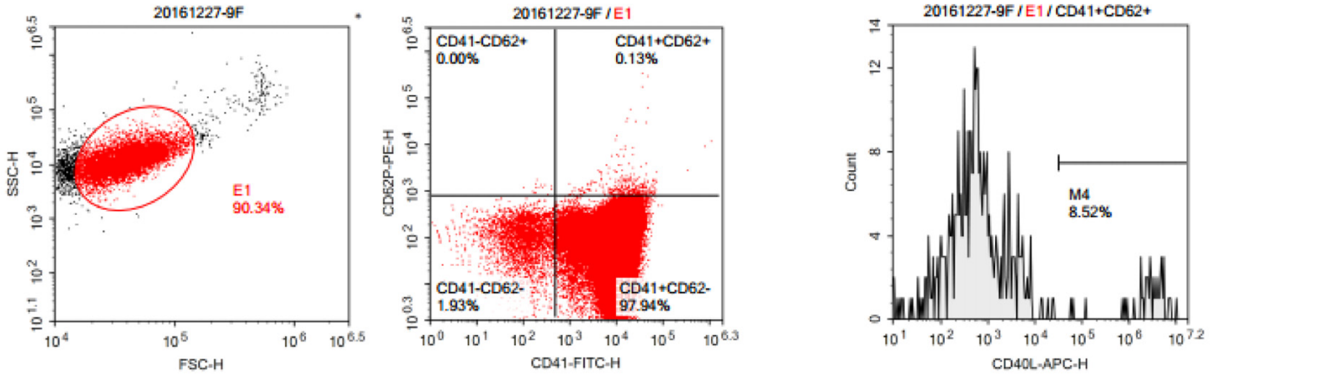

Gate	Count	% All	Mean X	CV X
All	462,919	100.00%	48,535	139.25%
E1	418,213	90.34%	43,282	50.95%

Gate	Count	% E1	% All	Mean X	CV X
E1	418,213	100.00%	90.34%	10,403	54.43%
CD41-CD62+	17	0.00%	0.00%	160	143.05%
CD41+CD62+	528	0.13%	0.11%	25,861	235.74%
CD41-CD62-	8,062	1.93%	1.74%	8	6,978.62 %
CD41+CD62-	409,606	97.94%	88.48%	10,588	47.67%

Gate	Count	% CD41+CD62+	% All	Mean X	CV X
CD41+CD62+	528	100.00%	0.11%	296,376	388.83%
M4	45	8.52%	0.01%	3,465,251	62.42%

Supplementary Figure 2. Original figure of flow cytometry analysis on CD40L positive activated platelets. Panel 1.

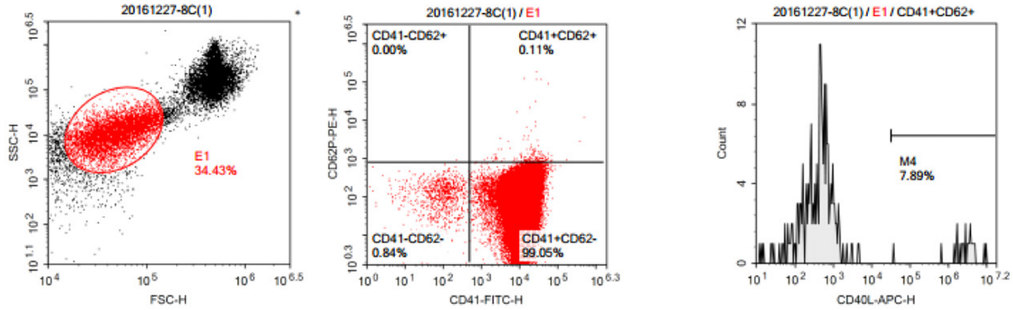

Gate	Count	% All	Mean X	CV X
All	797,295	100.00%	311,713	79.44%
E1	274,538	34.43%	56,323	52.54%

Gate	Count	% E1	% All	Mean X	CV X
E1	274,538	100.00%	34.43%	12,864	49.17%
CD41-CD62+	1	0.00%	0.00%	227	0.00%
CD41+CD62+	304	0.11%	0.04%	27,004	137.59%
CD41-CD62-	2,310	0.84%	0.29%	-430	-327.11%
CD41+CD62-	271,923	99.05%	34.11%	12,961	47.00%

Gate	Count	% CD41+CD62+	% All	Mean X	CV X
CD41+CD62+	304	100.00%	0.04%	267,726	411.78%
M4	24	7.89%	0.00%	3,386,934	65.94%

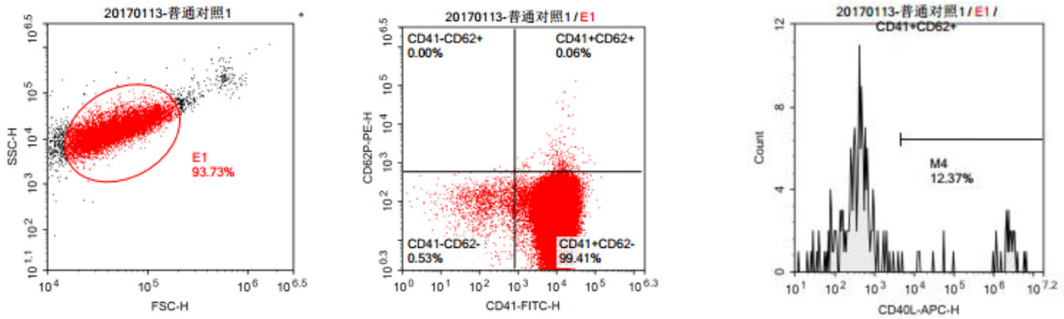

Gate	Count	% All	Mean X	CV X
All	528,013	100.00%	64,277	119.20%
E1	494,906	93.73%	56,700	61.22%

Gate	Count	% E1	% All	Mean X	CV X
E1	494,906	100.00%	93.73%	12,112	54.61%
CD41-CD62+	11	0.00%	0.00%	452	71.00%
CD41+CD62+	291	0.06%	0.06%	15,050	75.18%
CD41-CD62-	2,638	0.53%	0.50%	212	131.93%
CD41+CD62-	491,966	99.41%	93.17%	12,174	53.97%

Wild+BAPN

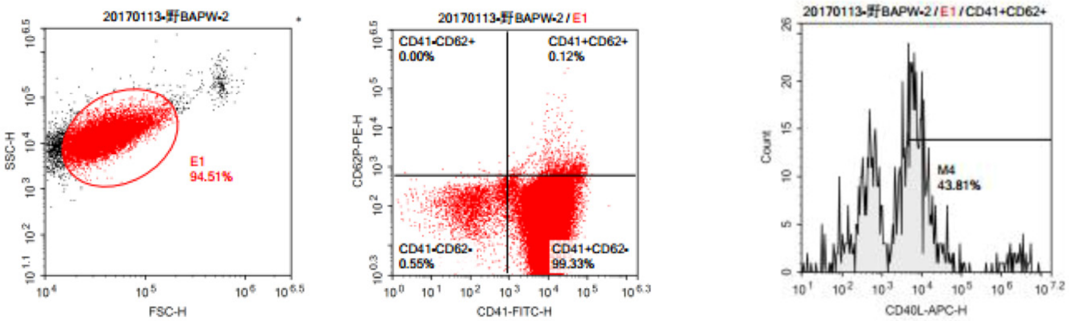

Gate	Count	% All	Mean X	CV X
All	985,067	100.00%	48,039	123.69%
E1	930,939	94.51%	43,706	52.63%

Gate	Count	% E1	% All	Mean X	CV X
E1	930,939	100.00%	94.51%	10,742	51.64%
CD41-CD62+	23	0.00%	0.00%	299	115.26%
CD41+CD62+	1,098	0.12%	0.11%	28,195	76.57%
CD41-CD62-	5,135	0.55%	0.52%	147	162.83%
CD41+CD62-	924,683	99.33%	93.87%	10,781	50.33%

Gate	Count	% CD41+CD62+	% All	Mean X	CV X
CD41+CD62+	1,098	100.00%	0.11%	125,019	524.73
M4	481	43.81%	0.05%	284,053	341.04

Supplementary Figure 2. Original figure of flow cytometry analysis on CD40L positive activated platelets. Panel 2.

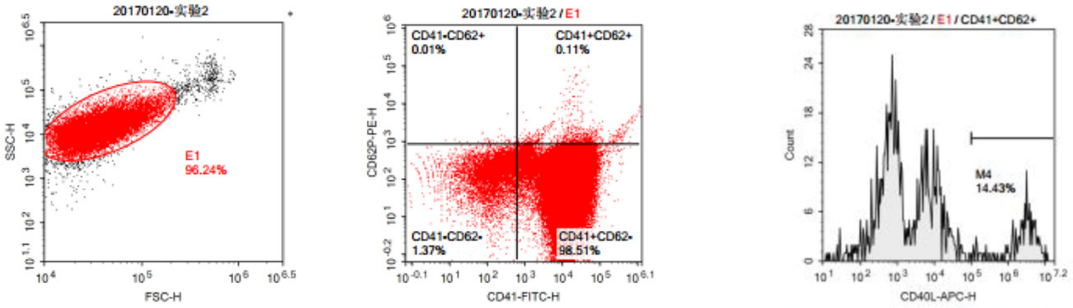

Gate	Count	% All	Mean X	CV X
All	1,023,469	100.00%	53,755	130.94%
E1	984,959	96.24%	45,306	66.22%

Gate	Count	% E1	% All	Mean X	CV X
E1	984,959	100.00%	96.24%	13,614	77.65%
CD41-CD62+	80	0.01%	0.01%	165	145.49%
CD41+CD62+	1,109	0.11%	0.11%	69,451	246.91%
CD41-CD62-	13,489	1.37%	1.32%	121	159.79%
CD41+CD62-	970,281	98.51%	94.80%	13,739	62.50%

Gate	Count	% CD41+CD62+	% All	Mean X	CV X
CD41+CD62+	1,109	100.00%	0.11%	469,103	305.10%
M4	160	14.43%	0.02%	3,220,022	72.00%

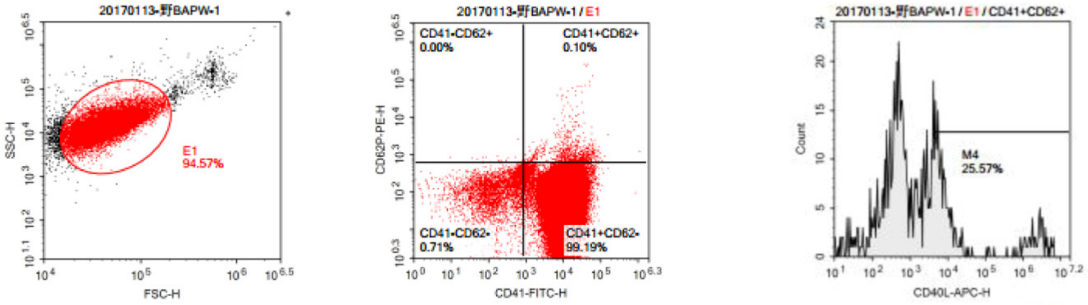

Gate	Count	% All	Mean X	CV X
All	1,074,215	100.00%	54,853	123.77%
E1	1,015,866	94.57%	48,386	54.47%

Gate	Count	% E1	% All	Mean X	CV X
E1	1,015,866	100.00%	94.57%	11,980	55.22%
CD41-CD62+	36	0.00%	0.00%	222	150.30%
CD41+CD62+	1,009	0.10%	0.09%	22,579	170.74%
CD41-CD62-	7,195	0.71%	0.67%	190	139.75%
CD41+CD62-	1,007,626	99.19%	93.80%	12,054	53.46%

Gate	Count	% CD41+CD62+	% All	Mean X	CV X
CD41+CD62+	1,009	100.00%	0.09%	186,390	421.11%
M4	258	25.57%	0.02%	726,444	195.79%

CD40L-/-

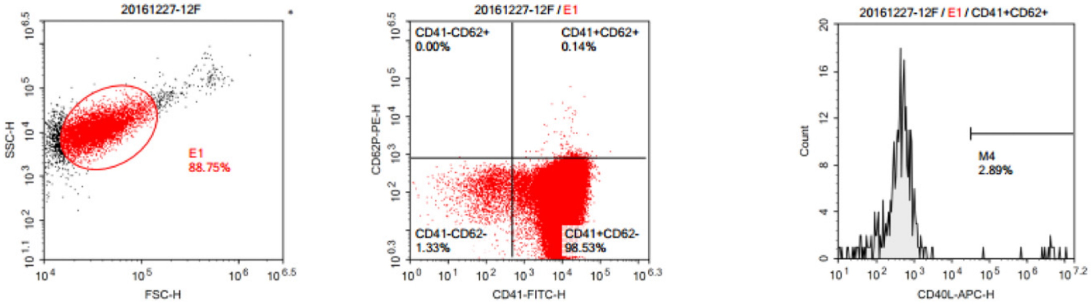

Gate	Count	% All	Mean X	CV X
All	372,695	100.00%	45,129	145.18%
E1	330,761	88.75%	38,686	54.03%

Gate	Count	% E1	% All	Mean X	CV X
E1	330,761	100.00%	88.75%	9,955	57.64%
CD41-CD62+	1	0.00%	0.00%	21	0.00%
CD41+CD62+	450	0.14%	0.12%	21,255	64.54%
CD41-CD62-	4,415	1.33%	1.18%	-213	-416.30%
CD41+CD62-	325,895	98.53%	87.44%	10,077	55.74%

Gate	Count	% CD41+CD62+	% All	Mean X	CV X
CD41+CD62+	450	100.00%	0.12%	125,388	681.06%
M4	13	2.89%	0.00%	4,328,752	63.59%

Supplementary Figure 2. Original figure of flow cytometry analysis on CD40L positive activated platelets. Panel 3.

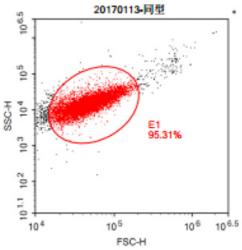

Gate	Count	% All	Mean X	CV X
All	491,075	100.00%	60,536	180.10%
E1	468,068	95.31%	53,613	53.21%

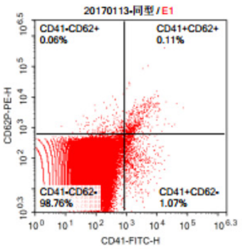

Gate	Count	% E1	% All	Mean X	CV X
E1	468,068	100.00%	95.31%	1,933	2,084.69 %
CD41-CD62+	292	0.06%	0.06%	382	77.77%
CD41+CD62+	505	0.11%	0.10%	3,314	153.17%
CD41-CD62-	462,265	98.76%	94.13%	66	168.40%
CD41+CD62-	5,006	1.07%	1.02%	174,276	200.22%

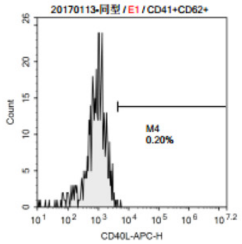

Gate	Count	% CD41+CD62+	% All	Mean X	CV X
CD41+CD62+	505	100.00%	0.10%	1,019	72.75%
M4	1	0.20%	0.00%	5,448	0.00%

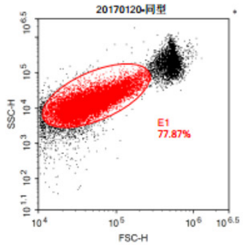

Gate	Count	% All	Mean X	CV X
All	1,126,200	100.00%	134,744	134.98%
E1	876,993	77.87%	56,168	70.45%

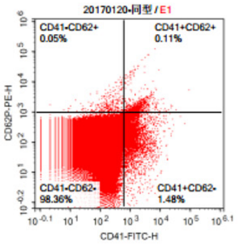

Gate	Count	% E1	% All	Mean X	CV X
E1	876,993	100.00%	77.87%	1,574	2,573.95 %
CD41-CD62+	407	0.05%	0.04%	227	81.77%
CD41+CD62+	970	0.11%	0.09%	2,352	174.28%
CD41-CD62-	862,609	98.36%	76.59%	67	168.67%
CD41+CD62-	13,007	1.48%	1.15%	101,517	312.39%

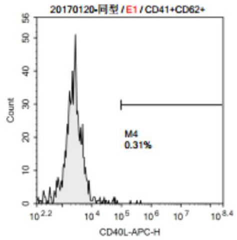

Gate	Count	% CD41+CD62+	% All	Mean X	CV X
CD41+CD62+	970	100.00%	0.09%	4,573	436.12%
M4	3	0.31%	0.00%	333,137	35.43%

CD40L-/- + BAPN

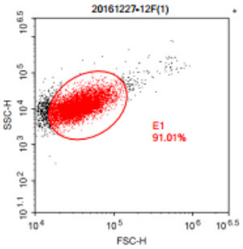

Gate	Count	% All	Mean X	CV X
All	353,312	100.00%	42,023	112.13%
E1	321,563	91.01%	39,385	48.97%

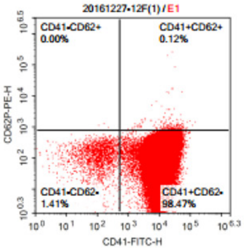

Gate	Count	% E1	% All	Mean X	CV X
E1	321,563	100.00%	91.01%	11,160	53.59%
CD41-CD62+	5	0.00%	0.00%	-36	-1,148.45%
CD41+CD62+	373	0.12%	0.11%	26,455	69.96%
CD41-CD62-	4,545	1.41%	1.29%	-42	-1,388.61%
CD41+CD62-	316,640	98.47%	89.62%	11,303	51.45%

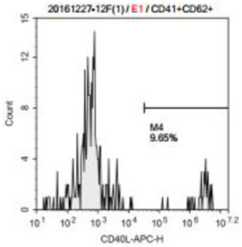

Gate	Count	% CD41+CD62+	% All	Mean X	CV X
CD41+CD62+	373	100.00%	0.11%	289,356	339.00%
M4	36	9.65%	0.01%	2,991,677	46.24%

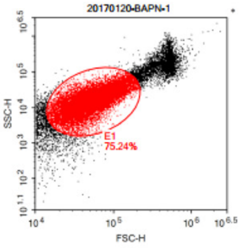

Gate	Count	% All	Mean X	CV X
All	1,224,325	100.00%	122,225	132.85%
E1	921,160	75.24%	59,655	62.35%

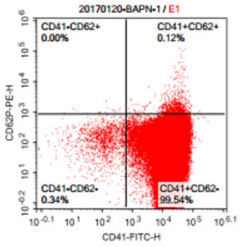

Gate	Count	% E1	% All	Mean X	CV X
E1	921,160	100.00%	75.24%	17,439	63.37%
CD41-CD62+	26	0.00%	0.00%	121	159.05%
CD41+CD62+	1,111	0.12%	0.09%	27,250	56.96%
CD41-CD62-	3,113	0.34%	0.25%	105	194.61%
CD41+CD62-	916,910	99.54%	74.89%	17,487	62.97%

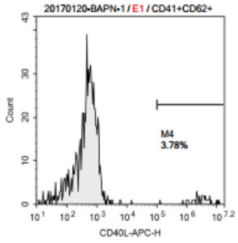

Gate	Count	% CD41+CD62+	% All	Mean X	CV X
CD41+CD62+	1,111	100.00%	0.09%	138,566	621.50%
M4	42	3.78%	0.00%	3,654,359	71.98%

Supplementary Figure 2. Original figure of flow cytometry analysis on CD40L positive activated platelets. Panel 4.

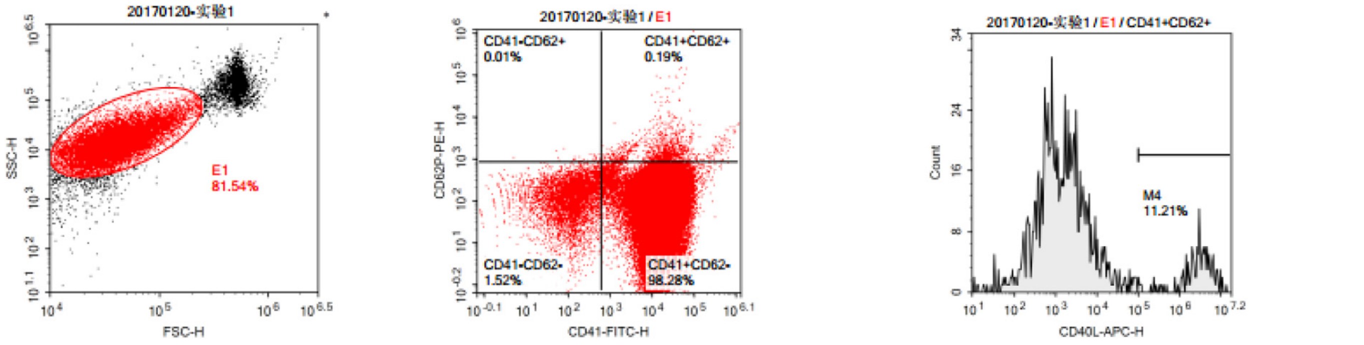

Gate	Count	% All	Mean X	CV X
All	927,340	100.00%	113,980	148.44%
E1	756,155	81.54%	48,406	70.86%

Gate	Count	% E1	% All	Mean X	CV X
E1	756,155	100.00%	81.54%	14,789	79.38%
CD41-CD62+	58	0.01%	0.01%	245	99.49%
CD41+CD62+	1,418	0.19%	0.15%	50,837	248.53%
CD41-CD62-	11,514	1.52%	1.24%	104	155.49%
CD41+CD62-	743,165	98.28%	80.14%	14,948	68.20%

Gate	Count	% CD41+CD62+	% All	Mean X	CV X
CD41+CD62+	1,418	100.00%	0.15%	409,399	343.71%
M4	159	11.21%	0.02%	3,620,609	68.04%

Supplementary Figure 2. Original figure of flow cytometry analysis on CD40L positive activated platelets. Panel 5. CD41+ indicates anti-CD41-FITC, CD62P+ indicates anti-CD62P-PE and CD41+ CD62P+ platelets indicates activated platelets. M4 means percentage of CD41+ CD62P+CD40L+ platelets divided by CD41+ CD62P+ platelets.
